# Knowledge, Attitudes, Practices and Zoonotic Risk Perception of Bovine Q Fever (*Coxiella burnetii*) among Cattle Farmers and Veterinary Personnel in Northern Regions of Cameroon

**DOI:** 10.3390/epidemiologia3040036

**Published:** 2022-10-26

**Authors:** Camille Teitsa Zangue, Justin Kouamo, Ferdinand Ngoula, Ludovic Pépin M’bapté Tawali, Moustapha Mohamed Fokom Ndebé, Dinayen Edwin Somnjom, Ranyl Noumedem Guefack Nguena, Mohamed Moctar Mouliom Mouiche

**Affiliations:** 1 School of Veterinary Medicine and Sciences, University of Ngaoundere, Ngaoundere P.O. Box 454, Cameroon; 2 Faculty of Agronomy and Agricultural Sciences, University of Dschang, Dschang P.O. Box 96, Cameroon; 3 Institute of Agricultural Research for Development, Polyvalent Station Bangangte, Bangangte P.O. Box 222, Cameroon; 4 Institute of Agricultural Research for Development, Polyvalent Station Bertoua, Bertoua P.O. Box 203, Cameroon; 5 National Veterinary Laboratory (LANAVET), Garoua P.O. Box 503, Cameroon

**Keywords:** bovine Q fever, cattle farmers, veterinary personnel, Cameroon

## Abstract

A cross-sectional survey was conducted to investigate the knowledge, attitudes, practices and zoonotic risk perception of Q fever among 484 selected cattle farmers (438) and veterinary personnel (46) in three northern regions of Cameroon. Data collection was conducted using questionnaires and responses were recoded into binary scale. An ANOVA test was used to assess significant differences in mean knowledge, attitude, practice and zoonotic risk perception (KAPP) scores between regions, while Linear regression was done to explore the relationship between demographic characteristic and KAPP. Overall, surveyed had low mean scores for knowledge (0.02 ± 0.11), desirable attitude (0.30 ± 0.16), appropriate practice (0.43 ± 0.13) and negative perception of zoonotic risks (0.05 ± 0.11). The means knowledge, attitude, practice and risks perception scores of cattle farmers were lower than those of veterinary personnel. The nature of respondent was negatively associated to knowledge and risks perception, while regions were negatively correlated to attitude and practice. These results revealed significant knowledge gaps, low levels of desired attitudes, and high-risk behavioral practices. To improve awareness, control programs are needed to update knowledge on medical personnel and to prevent animal-to-human transmission.

## 1. Introduction

Coxiellosis (Q fever) is a zoonosis that occurs in most countries. The causal agent is the obligate intracellular bacterium *Coxiella burnetii*, which displays different morphological forms in its developmental cycle [1]. Domestic ruminants are most important reservoir and major sources of human infection, where subclinical carriers shed the bacteria in various secretions and excreta [2]. Humans generally acquire infection through air-borne transmission and the clinical signs are polymorphic and nonspecific [3]. Acute Q fever in human most often results in a flu-like illness, hepatitis, or pneumonia, whereas chronic Q fever may develop in patients with predisposing factors, with most severe manifestation being endocarditis. The overall impact of *C. burnetii* infection on public health in the world is under control but there is a need for a better surveillance system. In human epidemic situations, active surveillance of acute Q fever is the best strategy for avoiding chronic cases [4].

The epidemiology of Q fever in Cameroon is poorly understood due to apparent negligence of the disease by both medical and veterinary personnel and the limited capacity to enable meaningful epidemiological surveys. Recent studies conducted in aborted dairy cows in Algeria [5], cattle from Ibarapa area of Nigeria [6] and slaughterhouse cattle of Jimma town, South Western Ethiopia [7] showed that diseases are present in many African countries. The lack of data on the bovine herd makes it difficult to manage Q fever in Cameroon. Little information is available about the epidemiology of bovine Q fever, but prevalence of 14.6% (CI 11.8–18.0%) and 12.4% (CI 9.6–15.9%) have been reported in North West region and Vina division of Adamawa, Cameroon, respectively [8].

In Cameroon, cattle herds was estimated about 8 million heads in 2018 and more than 80% of the national cattle livestock population comes from northern regions. The most practiced farming system is extensive which promotes close and prolonged contact between humans and cattle, and increase risk of zoonotic disease transmission [9]. Livestock farmers and veterinarians are considered high-risk groups, as they are more exposed to diseases transmitted either through direct contact with infected animals or materials [10]. There is very limited information on the knowledge, attitudes, practices and risks perception (KAPP) of cattle farmers and veterinary personnel about Q fever transmitted by cattle in Cameroon, and their implication is primordial for the successful control and management of the spread of Q fever.

## 2. Materials and Methods

### 2.1. Study Area Description

**Figure 1:**
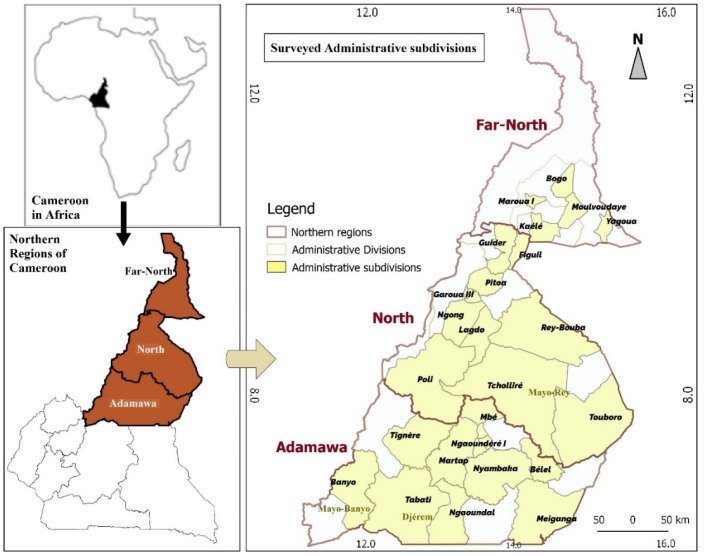
Map of study area.

The study was conducted in Adamawa, North and Far regions of Cameroon (Figure 1 and Table 1).

### 2.2. Study Design and Sampling

A semi-structured questionnaire was the main tool used to collect data between July 2020 to December 2021. The scientific research and ethics committee of School of Veterinary Medicine and Sciences of the University of Ngaoundere-Cameroon (2020/164/UN/R/VRE-PDTIC/DAAC/D-ESMV/DAACRS) provided ethical approval for this research. After taking verbal consent from respondents a standardized questionnaire was used to gather information regarding cattle farmers and veterinary personnel knowledges, attitudes, practices and perception of the risks about Q fever and its zoonotic potential. Demographic characteristics of cattle farmers and veterinary personnel with respect to gender, age, education, training and experience in cattle farming were collected. The questionnaire was administered in French and translated into native language at the time of the interview to minimize confusion and maximize responses accuracy, and focused on specific issues for each category of respondents [11].

### 2.3. Sampling Procedures

A minimum sample size of 370 was estimated [12], based on previous reports on the estimate of 39% Q fever prevalence reported in Adamawa region of Cameroon [13] with a confidence interval of 95% and precision of 5%. Random number generation technique was used for selection of cattle farmers and veterinary personnel obtained from a list of the Regional Delegations of Livestock, Ficheries and Animal Industries (RDLFAI) of the Northern Regions of Cameroon. Cattle farms with abortions in last two years were included in the study. A stratified random technique was used, and cattle farmers of each region were sampled according to the proportion of national flock size. Veterinary personnel of each region was randomly selected based on their activity in cattle farms and management of abortion at pass. A total of 484 respondents provided complete information during the interview of which 438 (90.50%) were cattle farmers and 46 (9.50%) were veterinary personnel.

### 2.4. Data Analysis

The data collection was conducted using questionnaires administered through interview and responses were recoded into binary scale. Each correct answer was credited 1 point representing sufficient knowledge, desirable attitude, appropriate practice towards Q fever and positive risk perception, while 0 represented insufficient knowledge, undesirable attitude, inappropriate practice and negative perception of risk. Responses such as “indifferent” or “not know” were coded as undesirable, inappropriate and negative. Sufficient answers were coded 1 and insufficient 0 for open items. The sum of sufficient responses provided by each participant divided by total number of items within the category gave a percentage of correct answers. For questions with several choices the number of points was obtained by dividing the point corresponding to the question by number of choices. The results were then converted into a percentage for an overall assessment and interpretation was according to KAPP survey evaluation grid [14]. Descriptive statistics using Statistical Package for Social Sciences (SPSS) version 23.0 were used to indicate the demographic distribution of farmers, veterinary personnel and farm characteristics. One-Way ANOVA associated with post hoc Duncan tests analysis was used to assess significant differences of mean KAPP scores across regions. For dichotomous variables, an independent *t*-test was used for comparison. Mean ± Standard deviation was used to represent the level of knowledge, attitudes, practices and risk perception towards Q fever. The relationships between demographic characteristics and KAPP scales were explored with a linear regression model.

## 3. Results

### 3.1. Demographic Characteristics

Most of respondents included in the study were males (98.97%), aged between fifty-nine and seventy years (38.60%) and work force composed mainly of employees (98.35%). About 45.66% had no education, 84.30% had duration in cattle farming more than 30 years and 86.98% had no training in cattle farming. More than 97.31% of respondents reported the presence of ectoparasites in their environment and 99.38% implemented control measures. The main breed kept was Gudali (49.79%) and local livestock markets were the main places where the animals were bought (98.03%) and sold (96.02%). The farms raised cattle predominantly for meat consumption (77.12%), and 46.79% of farmers had one herd with average number of 77.76 ± 17.51 heads. Cattle farmers reported that 63.01% of the farms were in charge of veterinary staff but 94.02% come only in case of problems.

### 3.2. Knowledge of Q Fever

The distribution of knowledge of cattle farmers and veterinary personnel in northern regions are presented in Table 2. Overall, the means knowledge scores of Q fever in surveyed areas were 0.02 *±* 0.11. The study showed that the mean knowledge scores of cattle farmers were significantly lower than veterinary personnel. Age, educational level, regions, sex, training and duration in cattle farming were main factors influencing knowledge score (*p* < 0.05).

### 3.3. Attitudes and Practices toward Q Fever

**Figure 2:**
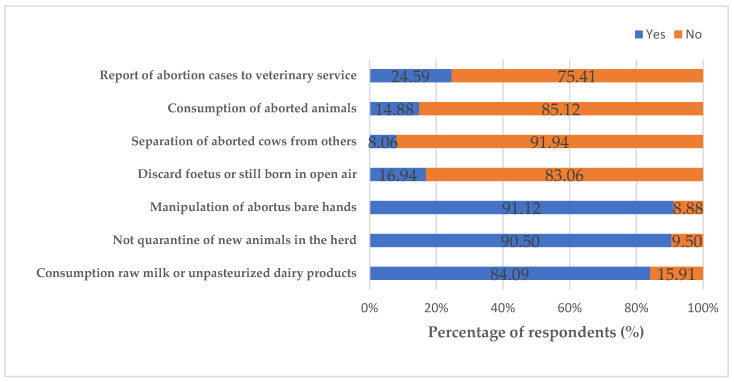
Distribution of criteria for evaluating abortion management and livestock husbandry attitudes and practices by cattle farmers and veterinary personnel in Adamawa, North and Far North Regions of Cameroon (*N* = 484).

Factors such as reporting of an abortion case to veterinary service, separating aborted cow from others, slaughtering aborted animal for consumption, fate of foetus or stillborn in open air, manipulating of abortus bare hands, consuming raw milk or unpasteurized dairy products and not quarantine for new animals in herd were used to assess inappropriate practices and attitudes of cattle farmers and veterinary personnel (Figure 2). The means of attitude and practices scores of Q fever were 0.30 ± 0.16 and 0.43 ± 0.13, respectively. Veterinary personnel had more appropriate attitudes and practices than cattle farmers (*p* < 0.05). Respondents with none or primary educational level with duration in cattle farming more than twenty years showed less appropriate attitudes and practices compared to others categories (*p* < 0.05). The distribution of mean scores of practices and attitudes of cattle farmers and veterinary personnel towards abortion management and livestock husbandry is presented in Table 3.

### 3.4. Risks Perception of Q Fever

As for perception of risks by interviewed, a means scores of 0.05 *±* 0.11 was observed. Cattle farmers were less sensitized than veterinary personnel (*p* < 0.05). Respondents aged between twenty-six and thirty-six years with secondary or higher educational levels were more aware compared to others categories (*p* < 0.05). However, duration in cattle farming (*p* = 0.0005), sex (*p* = 0.04) and training in cattle farming (*p* = 0.00001) significantly influenced risk perception of cattle farmers and veterinary personnel in the said localities (Table 4).

### 3.5. Measures of the Level of KAPP Association in Different Study Regions

Table 5 and Table 6 showed the level of KAPP association of with demographic characteristics of respondents in a Linear regression model analysis at *p* < 0.05 and *p* < 0.01. The nature of respondent was negatively associated to knowledge (6% decrease) and risks perception (5% decrease) while locality was negatively correlated to attitude (2% decrease) and practice (2% decrease). Educational level and training in cattle farming were positively associated to knowledge, attitude, practices and risks perception of respondents. Sex was negatively associated to attitude, practices (15% decrease) and perception of risks (13% decrease).

## 4. Discussion

This study investigated cattle farmer and veterinary personnel knowledge, attitudes, practices and risks perception about Q fever in Adamawa, North and Far North Regions of Cameroon. The result revealed low overall knowledge of Q fever, inadequate attitude with inappropriate practice, and negative risk perception of respondents. The study showed that cattle farmers have low knowledge of Q fever, which was associated to higher proportion of respondents without education and training in cattle farming. These results are in agreement with study undertaken in Tajikistan [15] and in Adamawa, littoral, west and Centre Regions of Cameroon [16]. In contrast to this finding, a study in turkey showed that awareness of zoonotics diseases among cattle farmers were positively associated to higher educational level [17]. The main cattle breeding system in Cameroon being transhumence, the farmers live in fairly remote rural areas and therefore do not have access to information. The lack of familiarity with Q fever can be due to absence of a specific word for disease in local dialects. Veterinary personnel showed lower scores of knowledges in general, but nurse and technicians were least and veterinary doctors were most knowledgeable about Q fever. Same was observed in Northern Regions of Uganda [18]. This could be attributed to their professionnal training because veterinary doctors are thought to be knowledgeable on issues of this disease awing to their professionnal training [19]. However, such poor knowledge among veterinary personnel in this study points to Q fever being an unknown and neglected disease in Cameroon thus its importance not emphasized during professional training in the country. Educational level and training in cattle farming have been positively associated to knowledge of Q fever. In addition to this, the increasing years of farming correlated with poor knowledge of the disease. These results are in contrast with those reported in Uganda [18], which revealed that accumulation in experience and insights about the disease occurs with age. But similar results were obtained in Greece [20], and can be attributed to improvements of education system across the years and acquaintance of new generation with technological developments.

The overall attitude and practices scores of respondents were average in cattle farmers and good in veterinary personnel. The means of attitudes of veterinary personnel were comparable but higher than cattle farmers. Incorrect attitudes towards prevention of Q fever disease from animal birth products, such as assisting and manipulation of abortus with bare hands, and improper disposal of aborted foetus and placenta, strongly support the need for culturally appropriate health education among cattle farmers and veterinary personnel in rural communities. The pollution of the environment with abortus or cadavers by some farmers and veterinary personnel demonstrates lack of awareness of the risk associated with managing abortions in livestock [21]. Attitude and practices of respondent were positively and significantly correlated to higher educational level and training in cattle breeding. This study showed that farmers who are monitored by animal health staff develop better behavioral practices in managing abortions on their farms. In this extensive farming system, majority of farmers buy drugs to treat their animals in case of illness and call vet only in case of failure or when symptoms worsen. The education campaign or support of veterinary personnel could enable farmers to be more efficient and vigilant about their actions in farms. Therefore, it is urgent to educate entire communities to proper handling of aborted foetuses and placenta to reduce environmental transmission of Q fever. It is important to change attitude of the community to improve their behavioural practices towards zoonotic diseases transmission and prevention [22]. Concerning utilization of protective gloves and masks when handling an abortion, it was limited to 5.48% of cattle farmers and 41.30% of veterinary personnel. The prevailing poverty in these regions, the poor knowledge with this practice and lack of access to protective clothing like gloves can explain this observation. This result corroborates with those who reported that only 31% of farmers use professional protective clothing when handling an abortion, and this negligence is an enhancing factor for microbial contamination especially by respiratory, ocular and oral routes, since most abortifacients are the causative agents of highly pathogenic zoonoses [23]. Livestock authorities should ensure that proper protrective gloves are used by all livestock handlers as first line of defense. This study futher identified certains practices like consuming raw milk or unpasteurized dairy products and no quarantine for new animals in herd that can promote Q fever transmission within human population if animals are infected [24]. Comparable result were reported in Kenya [16] and Pakistan [25]. To improve Q fever awareness, vaccination of herds and cattle testing would generate a unique opportunity [26].

Despite the fact that majority of respondents had undesirable attitudes and inappropriate practices towards prevention of Q fever, veterinary personnel in general and doctors in particular perceived this disease as a risk of public health unlike cattle farmers. This observation further demonstrates the importance of continuous education and awareness among farmers and veterinary personnel on importance of improving their knowledge, adopting good attitude and practices in livestock farming in order to improve the profitability of this speculation [27]. Permanent communication should be made on zoonotic diseases and Q fever in particular, with populations at risk in regions with a high density of ruminants in Cameroon.

This study has certain limitations that must be taken into consideration when interpreting the results. Due to the nature of the data collection strategy, based on face-to-face participation, the response rate was high. Even if the questionnaire seemed hard to fill, the extent number of the items was helpful in capturing more responses regarding farmers’ and veterinary personnel behavior towards Q fever, practice, attitude and risk perception. The assessments of attitudes and practices toward Q fever and vector control have relied on self-reported data collected through interviews and could potentially be affected by social desirability bias. However, the low scores obtained by the majority of the participants indicate that this may not be so.

## 5. Conclusions

This study assessed knowledge, attitudes, practices and risk perception associated with bovine Q fever (*Coxiella burnetii*) on cattle farmers and veterinary personnel in Northern Regions of Cameroon. The KAPP tool used revealed low overall knowledge, inappropriate attitudes, inadequate practices and negative risk perception by cattle farmers more than veterinary personnel. Veterinary doctors showed better knowledge, attitude, practice and zoonotic risk perception than nurses and technicians. Overall, respondents with none or primary educational level, with duration in cattle farming more than twenty years showed less knowledge, attitude, practice and zoonotic risk perception of Q fever. Risk perceptions of Q fever influence overall attitudes and practices towards the disease in study respondents. The identified risk factors require the implementation of education forums aimed at equipping cattle farmers and veterinary personnel with operating procedures to help prevent the transmission of this emerging zoonotic infectious disease. Community health education programs need for targeted to minimize the transmission of zoonotic pathogens from abortion products of bovine during manipulation. There is need to undertake sensitization efforts to update knowledge and organize vaccination campaign as well as strengthen One -Health collaborations in order to effectively mitigate the zoonotic threats of this disease.

## Figures and Tables

**Table 1:** Study area characteristics [9].

Characteristics	Regions		
	Adamawa	North	Far North
Latitude	5–8° N	8–10° N	10–12° N
Longitude	11–14° E	12–14° E	14–15° E
Surface Area (km^2^)	63,701	66,090	34,263
Climate	Sudano Guinean	Soudanese	Sudano Sahelian
Mean annual temperature (°C)	22–25	21–34	25–35
Mean annual precipitation (mm)	900–1500	800–900	600–1000
Estimated population in 2012 (inhabitants)	1,080,500	1,687,959	3,111,792
Population density (inhabitant/km^2^)	17	26	91

mm—millimeters; km^2^—Square kilometer; °C—Celsius degree; N—North; E—East.

**Table 2:** Distribution of mean knowledge scores of Q fever according to demographic characteristics (*N* = 484) in Adamawa, North and Far North Regions of Cameroon.

Factor	Variables	*N*	Knowledge	
			Mean ± SD	*p* Value
Respondents	Cattle farmers	438	0.004 ± 0.04 ^a^	0.001
	Veterinary doctors	16	0.38 ± 0.25 ^b^	
	Veterinary nurses	24	0.10 ± 0.23 ^c^	
	Veterinary technicians	6	0.17 ± 0.27 ^c^	
Age range (Year)	26–36	17	0.18 ± 0.23 ^a^	0.02
	37–47	99	0.01 ± 0.08 ^b^	
	48–58	181	0.02 ± 0.12 ^b^	
	59–70	187	0.01 ± 0.08 ^b^	
Educational level	None	221	0.002 ± 0.03 ^a^	0.00005
	Primary	199	0.004 ± 0.04 ^a^	
	Secondary	37	0.10 ± 0.22 ^b^	
	Higher	27	0.22 ± 0.27 ^b^	
Regions	Adamawa	241	0.02 ± 0.11 ^a^	0.02
	North	122	0.04 ± 0.13 ^a^	
	Far North	121	0.007 ± 0.06 ^b^	
Duration in cattle farming (years)	<10	6	0.22 ± 0.25 ^a^	0.04
	10–20	24	0.11 ± 0.20 ^a^	
	21–30	46	0.03 ± 0.13 ^b^	
	>30	408	0.01 ± 0.09 ^b^	
Sex	Male	479	0.02 ± 0.10 ^a^	0.001
	Female	5	0.18 ± 0.25 ^b^	
Training in cattle farming	Yes	64	0.15 ± 0.24 ^a^	0.0004
	No	420	0.004 ± 0.04 ^b^	

Values within a column with ^a^, ^b^ and ^c^ differ significantly at *p* < 0.05. SD: Standard deviation.

**Table 3:** Distribution of mean attitude and practice scores towards abortion management and livestock husbandry according to demographic characteristics (*N* = 484) in Adamawa, North and Far North Regions of Cameroon.

Factor	Variables	*N*	Attitude		Practices	
			Mean ± SD	*p* Value	Mean ± SD	*p* Value
Respondents	Cattle farmers	438	0.27 ± 0.12 ^a^	0.01	0.41 ± 0.11 ^a^	0.004
	Veterinary doctors	16	0.69 ± 0.13 ^b^		0.64 ± 0.15 ^b^	
	Veterinary nurses	24	0.61 ± 0.11 ^b^		0.57 ± 0.10 ^b^	
	Veterinary technicians	6	0.55 ± 0.16 ^b^		0.59 ± 0.17 ^b^	
Age range (Year)	26–36	17	0.48 ± 0.28 ^a^	0.001	0.53 ± 0.23	0.09
	37–47	99	0.34 ± 0.17 ^a^		0.44 ± 0.12	
	48–58	181	0.30 ± 0.16 ^b^		0.42 ± 0.13	
	59–70	187	0.27 ± 0.14 ^b^		0.42 ± 0.11	
Educational level	None	221	0.25 ± 0.11 ^a^	0.00002	0.40 ± 0.11 ^a^	0.0007
	Primary	199	0.28 ± 0.13 ^a^		0.42 ± 0.11 ^a^	
	Secondary	37	0.48 ± 0.19 ^b^		0.50 ± 0.16 ^b^	
	Higher	27	0.64 ± 0.14 ^c^		0.60 ± 0.14 ^c^	
Regions	Adamawa	241	0.32 ± 0.17	0.09	0.44 ± 0.13 ^a^	0.001
	North	122	0.27 ± 0.16		0.39 ± 0.12 ^b^	
	Far North	121	0.30 ± 0.15		0.44 ± 0.12 ^a^	
Duration in cattle farming (years)	<10	6	0.53 ± 0.27 ^a^	0.004	0.59 ± 0.23 ^a^	0.02
	10–20	24	0.44 ± 0.19 ^a^		0.51 ± 0.14 ^a^	
	21–30	46	0.31 ± 0.18 ^b^		0.42 ± 0.15 ^b^	
	>30	408	0.28 ± 0.15 ^b^		0.42 ± 0.12 ^b^	
Sex	Male	479	0.30 ± 0.16 ^a^	0.006	0.42 ± 0.12 ^a^	0.002
	Female	5	0.68 ± 0.16 ^b^		0.69 ± 0.23 ^b^	
Training in cattle farming	Yes	64	0.50 ± 0.20 ^a^	0.0008	0.54 ± 0.14 ^a^	0.01
	No	420	0.27 ± 0.13 ^b^		0.41 ± 0.11 ^b^	

Values within a column with ^a^, ^b^ and ^c^ differ significantly at *p* < 0.05. SD: Standard deviation.

**Table 4:** Distribution of risk perception of Q fever according to demographic characteristics (*N* = 484) in Adamawa, North and Far North Regions of Cameroon.

Factor	Variables	*N*	Perception of Risk of Q Fever	
			Mean ± SD	*p* Value
Respondents	Cattle farmers	438	0.02 ± 0.05 ^a^	0.0003
	Veterinary doctors	16	0.42 ± 0.26 ^b^	
	Veterinary nurses	24	0.17 ± 0.19 ^c^	
	Veterinary technicians	6	0.21 ± 0.28 ^bc^	
Age range (Year)	26–36	17	0.22 ± 0.28 ^a^	0.03
	37–47	99	0.04 ± 0.09 ^b^	
	48–58	181	0.04 ± 0.12 ^b^	
	59–70	187	0.03 ± 0.08 ^b^	
Educational level	None	221	0.02 ± 0.05 ^a^	0.001
	Primary	199	0.02 ± 0.05 ^a^	
	Secondary	37	0.13 ± 0.21 ^b^	
	Higher	27	0.28 ± 0.26 ^b^	
Regions	Adamawa	241	0.05 ± 0.12	0.27
	North	122	0.03 ± 0.07	
	Far North	121	0.04 ± 0.14	
Duration in cattle farming (years)	<10	6	0.29 ± 0.32 ^a^	0.0005
	10–20	24	0.13 ± 0.21 ^b^	
	21–30	46	0.05 ± 0.15 ^c^	
	>30	408	0.04 ± 0.09 ^c^	
Sex	Male	479	0.04 ± 0.11 ^a^	0.04
	Female	5	0.28 ± 0.32 ^b^	
Training in cattle farming	Yes	64	0.18 ± 0.25 ^a^	0.00001
	No	420	0.02 ± 0.05 ^b^	

Values within a column with ^a^, ^b^ and ^c^ differ significantly at *p* < 0.05. SD: Standard deviation.

**Table 5:** Linear regression model of knowledge and risk perception measures versus demographic characteristics of cattle farmers and veterinary personnel (*N* = 484) in Adamawa, North and Far North regions of Cameroon.

Factors	Knowledge Estimates (95% CI)	Perception of Risk of Q Fever Estimates (95% CI)
Respondents	−0.06 ** (−0.09; −0.04)	−0.05 ** (−0.08; −0.03)
Regions	0.005 (−0,005; 0.01)	−0.003 (−0.01; 0.007)
Age range (Year)	0.004 (−0,007; 0.01)	0.0001 (−0.01; 0.01)
Educational level	0.03 ** (0.02; 0.05)	0.04 ** (0.03; 0.06)
Sex	−0.07 (−0.16; 0.02)	−0.13 ** (−0.22; −0.04)
Duration in cattle farming (years)	−0.007 (−0.02; 0.004)	−0.002 (−0.01; 0.009)
Training in cattle farming	0.10 ** (0.07; 0.13)	0.10 ** (0.07; 0.13)
Constant	0.13 (−0.07; 0.32)	0.24 * (0.03; 0.45)
*N* = 484	R^2^ = 0.03	R^2^ = 0.03

** *p* < 0.01; * *p* < 0.05; CI: Confidence Interval.

**Table 6:** Linear regression model of attitudes and practices measures versus demographic characteristics (*N* = 484) in Adamawa, North and Far North regions of Cameroon.

Factors	Attitude Estimates (95% CI)	Practices Estimates (95% CI)
Respondents	0.01 (−0.02; −0.05)	−0.001 (−0.03; 0.03)
Regions	−0.02 ** (−0.03; −0.006)	−0.02 ** (−0.03; 0.008)
Age range (Year)	−0.009 (−0.03; −0.006)	−0.003 (−0.01; 0.01)
Educational level	0.07 ** (0.06; 0.09)	0.03 ** (0.02; 0.05)
Sex	−0.15 * (−0.27; −0.03)	−0.15 ** (−0.26; −0.04)
Duration in cattle farming (years)	−0.0008 (−0.02; 0.02)	0.002 (−0.01; 0.02)
Training in cattle farming	0.11 ** (0.07; 0.16)	0.07 ** (0.03; 0.11)
Constant	0.38 ** (0.10; 0.66)	0.62 ** (0.38; 0.87)
*N* = 484	R^2^ = 0.04	R^2^ = 0.019

** *p* < 0.01; * *p* < 0.05; CI: Confidence Interval.

## Data Availability

The data presented in this article are not available publicly due to ethical reasons and are available from the corresponding author upon reasonable request.
